# L-F001, a Multifunctional Fasudil-Lipoic Acid Dimer Prevents RSL3-Induced Ferroptosis *via* Maintaining Iron Homeostasis and Inhibiting JNK in HT22 Cells

**DOI:** 10.3389/fncel.2022.774297

**Published:** 2022-03-31

**Authors:** Weijia Peng, Ying Ouyang, Shuyi Wang, Jiawei Hou, Zeyu Zhu, Yang Yang, Ruiyu Zhou, Rongbiao Pi

**Affiliations:** ^1^School of Pharmaceutical Sciences, Sun Yat-sen University, Guangzhou, China; ^2^Sun Yat-sen Memorial Hospital, Sun Yat-sen University, Guangzhou, China; ^3^School of Medicine, Sun Yat-sen University, Guangzhou, China

**Keywords:** L-F001, ferroptosis, iron homeostasis, lipid peroxidation, c-Jun N-terminal kinase

## Abstract

Ferroptosis, an iron-dependent form of non-apoptotic cell death, plays important roles in cerebral ischemia. Previously we have found that L-F001, a novel fasudil-lipoic acid dimer with good pharmacokinetic characters has good neuroprotection against toxin-induced cell death *in vitro* and *in vivo*. Here, we investigated the protective effects of L-F001 against a Glutathione peroxidase 4 (GPX4) inhibitor Ras-selective lethality 3 (RSL3) -induced ferroptosis in HT22 cells. We performed MTT, Transmission Electron Microscope (TEM), Western blot, and immunofluorescence analyses to determine the protective effects of L-F001 treatment. RSL3 treatment significantly reduced HT22 cell viability and L-F001 significantly protected RSL3-induced cell death in a concentration-dependent manner and significantly attenuated Mitochondrial shrinkage observed by TEM. Meanwhile, L-F001 significantly decreased RSL3-induced ROS and lipid peroxidation levels in HT22 cells. Moreover L-F001could restore GPX4 and glutamate-cysteine ligase modifier subunit (GCLM) levels, and significantly deceased Cyclooxygenase (COX-2) levels to rescue the lipid peroxidation imbalance. In addition, FerroOrange fluorescent probe and Western blot analysis revealed that L-F001 treatment decreased the total number of intracellular Fe^2+^ and restore Ferritin heavy chain 1 (FTH1) level in RSL3-induced HT22 cells. Finally, L-F001 could reduce RSL3-induced c-Jun N-terminal kinase (JNK) activation, which might be a potential drug target for LF-001. Considering that L-F001 has a good anti-ferroptosis effect, our results showed that L-F001 might be a multi-target agent for the therapy of ferroptosis-related diseases, such as cerebral ischemia.

## Introduction

Cerebral ischemia, occurring as the result of cardiac arrest, brain injury, shock, and age-related diseases, and then leading to neuronal death and neurological deficits, is a leading cause of disability and death worldwide (Madl and Holzer, 2004). Clinically, there is almost no effective therapy except to restore the blood flow either by pharmacological or mechanical thrombolysis till now (Sarkar et al., 2019). However, only less than 10% of cerebral ischemia patients are eligible for tissue plasminogen activator therapy, and half of those patients fail to demonstrate clinical improvement (DeSai and Hays Shapshak, 2021). So, discovering new drugs against cerebral ischemia is still an urgent issue.

Ferroptosis, a newly discovered iron-associated programmed cell death was reported the play important role in cerebral ischemia. In terms of morphology, the density of mitochondria in ferroptosis is lower, and mitochondrial crests are reduced or disappeared and the outer mitochondrial membrane is ruptured (Dixon et al., 2012; Xie et al., 2016). The biochemical signs of ferroptosis are lipid peroxidation and intracellular elevated Fe^2+^ level (Xie et al., 2016). Meanwhile, the reduced Glutathione (GSH), Glutathione peroxidase 4 (GPX4) inactivation and COX-2 over activation also predict the occurrence of ferroptosis (Cao and Dixon, 2016).

Growing evidence indicates that iron is a risk factor in the development of cerebral ischemia and ferroptosis is involved in the cerebral ischemia (She et al., 2020). The intracellular elevated Fe^2+^ level is increased in ischemic brains and iron chelation reduces ischemia-induced brain injury (Abdul et al., 2021). Moreover, several studies elucidated that the activation of ferroptosis following excitatory toxicity in cerebral ischemia induced by the lipid peroxidation mediated by the degradation of GPX4 and overexpression of Acyl-CoA Synthetase Long-Chain Family Member 4 (She et al., 2020; Cui et al., 2021). Meanwhile, the pathology of cerebral ischemia can be effectively alleviated by lipophilic ferroptosis inhibitors such as ferrostatin-1 and liproxstatin-1 (Alim et al., 2019; Guan et al., 2021). In addition, iron metabolism plays an important role in ferroptosis’s pathogenesis, which is also a potential target for cerebral ischemia and ischemic stroke (Dixon and Stockwell, 2014; Doll and Conrad, 2017; Chen et al., 2021). When iron homeostasis is broken, the iron redox couple (Fe^3+^/Fe^2+^) could trigger a Fenton reaction which causes overwhelming lipid peroxidation and definitively induces ferroptosis. And using the iron-chelating agent deferoxamine pretreatment can protect against both ferroptosis and cerebral ischemia (Li et al., 2008). Additionally, c-Jun N-terminal kinase (JNK), one member of the mitogen-activated protein kinase (MAPK) plays important role in regulating iron homeostasis through JNK/Sp1 and Stat4/Sp1 signal (Qiu et al., 2020).

**Figure 1:**
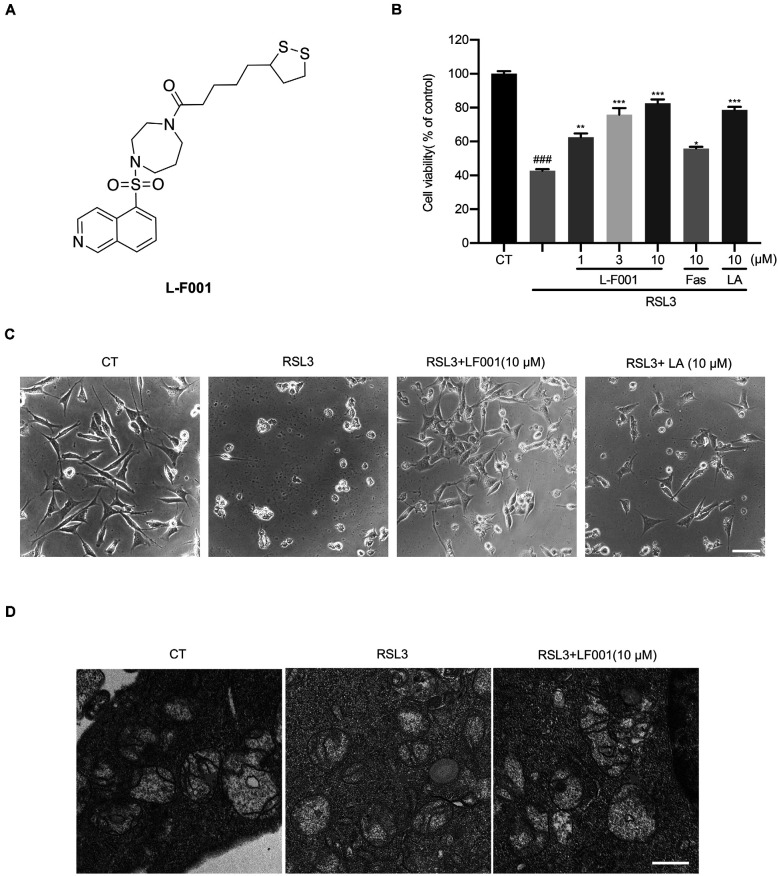
L-F001 reduces RSL3-induced cytotoxicity in HT22 cells. **(A)** Chemical structure of L-F001. **(B)** HT22 cells were treated with 0.1 μM RSL3 alone, and then added L-F001 (1–10 μM), fasudil (10 μM), and LA (10 μM) at 37°C for 24 h. MTT assays were used to determine cell viability (*n* = 5). **(C)** The morphology of HT22 cells (scale bar = 25 μm). **(D)** Transmission electron microscopy pictures of mitochondrial in cells from control, RSL3, and RSL3 + L-F001 group (Scale bar = 1,200 nm). ^###^*P* < 0.001 compared with the control group. *P < 0.05, **P < 0.01, and ***P < 0.001 compared with the RSL3-induced group.

L-F001 (Figure 1A), a fasudil-lipoic acid derivative synthesized by our lab has neuroprotection with multifunctional effects such as Rho-associated kinase (ROCK) inhibition. Our previous studies confirmed that L-F001 could prevent cell death induced by paraquat through alleviating endoplasmic reticulum stress and mitochondrial dysfunction in PC12 cells (Shen et al., 2015). In addition, L-F001 also could effectively reduce the cytotoxicity induced by 6-Hydroxydopamine hydrobromide (6-OHDA) *in vitro* and 1-methyl-4-phenyl-1,2,3,6-tetrahydropyridine (MPTP)-induced dopamine neuron toxicity in mice and improve the levels of p-Akt (Ser473), p-GSK3β(Ser9) to exert a neuroprotective effect (Luo et al., 2017). Moreover, L-F001 can significantly inhibit neuroinflammation induced by LPS and reduce microglial activation *in vitro* and *in vivo* (Chen et al., 2017). Considering ferroptosis plays important roles in these PD models (Tian et al., 2020; Mahoney-Sánchez et al., 2021) and L-F001 has anti-PD effects, we hypothesized that L-F001 might be benefit for ferroptosis to rescue other diseases, which might be a target for the therapy of cerebral ischemia. RSL3 is a GPX4 inhibitor which can block the ability for GPX4 that catalyzes GSH to oxidized glutathione (GSSG) and then reduces toxic peroxide to nontoxic hydroxyl compound to indirectly inhibits lipid peroxidation (Yang et al., 2014). Here we investigate the effects of L-F001 on RSL-3-induced ferroptosis in HT22 cells, a mice hippocampal cell line. Our data showed that L-F001 could inhibit RSL3-induced lipid peroxidation, maintain iron homeostasis, and inhibit the JNK pathway to attenuate ferroptosis. And L-F001 might be a multi-target agent for the therapy of ferroptosis-related diseases, such as cerebral ischemia.

## Materials and Methods

### Materials

L-F001 (purity>98%) was synthesized before, and the chemical characterization (mass spectroscopy, purity, etc) could be found over there (Chen et al., 2014). Dulbecco’s Modified Eagle’s Medium (DMEM) was from Gibco-BRL (NY, USA). Fetal bovine serum (FBS) was from Hyclone (Logan, UT, USA). A BCA protein assay kit was purchased from Thermo (Waltham, MA, USA). DMSO was obtained from Sigma-Aldrich Inc. (St Louis, MO, USA). Anti-GPX4 antibody, anti-FTH1antibody from Abcam (Cambridge, USA). Anti-COX-2, anti-JNK/p-JNK antibody from Cell Signaling Technology (Woburn, USA). Anti- GCLM antibody form ABclonal (Wuhan, Hubei, P.R.C.).

### Cell Culture

The sources of the cells are described in previous articles (Lu et al., 2020). HT22 cells were cultured in DMEM (10% FBS) under 5% CO_2_ and 37°C. And cells were subcultured every 2 days. Morphological change of induced cells was observed by phase-contrast microscopy (Olympus, Tokyo, Japan).

### MTT Assay

MTT assay was prepared according to our previous article to determine cell viability (Peng et al., 2021). HT22 cells were cultured in 96-well plates at 5 × 10^3^ cells per well and cultured 24 h before administration. Ten microliter of MTT (5 mg/ml) was then added to each well, and the mixture was incubated for 2 h at 37°C. MTT reagent was then carefully replaced with DMSO (100 μl per well) to dissolve formazan crystals. After the mixture was shaken at room temperature for 10 min, absorbance was determined at 490 nm using a microplate reader (Bio-Tek, USA). Cell survival assays were performed in triplicate.

### Transmission Electron Microscope

Transmission electron microscope protocol was performed as previously described (Peng et al., 2021). HT22 cells were grown on a 60 mm dish at 6 × 10^5^ cells per dish and cultured 24 h before administration. Then cells were treated with N2L and RSL3. A transmission electron microscope observed the ultrastructure of mitochondria. Briefly, HT22 cells were fixed following drug treatment with 4°C pre-cooled 2.5% glutaraldehyde for 24 h and with 1% osmium tetraoxide for 1 h. The cells were then dehydrated for 15 min at a series of acetone concentrations (50%, 70%, 80%, 90%, and 100%) and embedded in resin. The samples were sliced and double-stained with uranyl acetate and lead citrate, and representative images were obtained using a JEM-1400 electron microscope (JEOL Ltd., Japan).

### Measurement of ROS

Measurement of ROS was performed as previously described (Peng et al., 2021). The DHE probes were diluted 1:1,000 with serum-free medium to a final concentration of 10 μM. The serum-free cell culture medium was removed, and an appropriate volume of diluted DHE was added. The appropriate volume to cover the cells was added and the cells were incubated for 20 min in a 37°C cell incubator. The cells were washed three times with serum-free cell culture to adequately remove DHE that did not enter the cells. The high-content screening (HCS) system (Thermo, Waltham, MA, USA) was used to take fluorescence images and analyze the data (excitation wavelengths = 549 nm).

### Measurement of Lipid Oxidation Level

Measurement of lipid oxidation level was using BODIPY 581/591 C11 probe (Glpbio, Guangzhou, China). HT22 cells were grown on a 20 mm glass-bottom dish at 5 × 10^4^ cells per dish and cultured 24 h before administration. Cells were cultured with 2.5 μM C11-BODIPY581/591 for 30 min after drug treatment. The excitation and emission band of oxidized type is pass of 460–495 nm and 510–550 nm, respectively. But the excitation and emission band of reduced type is pass of 565–581 nm and 585–591 nm, respectively. Then photos were taken under a laser confocal microscope (Olympus, Tokyo, Japan).

### Measurement of Endogenous Hydroxyl Radicals

Rho-Bob is a gift from Prof. Fang Liu from Guangzhou University of Chinese Medicine, which detected the endogenous hydroxyl radicals in cells and the measurement was performed as previously described (Peng et al., 2021). Cells were cultured with 5 μM Rho-Bob for 30 min and observed under a confocal microscope. The excitation and emission band of the oxidized type is 532 nm. And the excitation and emission band of the reduced type is pass of 580–600 nm and 640–660 nm, respectively. Then photos were taken under a laser confocal microscope (Olympus, Tokyo, Japan).

### Detection of Intracellular Ferrous Ion Content

Cells were cultured with 1 μM FerroOrange for 30 min and observed under a confocal microscope (Ex/Em: 561 nm/570–620 nm).To facilitate the observation and analysis, the fluorescence signal from FerroOrange was marked as orange. And cell images were taken using a laser confocal microscope (Olympus, Tokyo, Japan).

### Immunofluorescence

Measurement of Immunofluorescence was performed as previously described (Chen et al., 2014). The cells were washed three times with PBS. After being fixed and blocked, the cells were stained overnight at 4°C with anti-COX-2 antibody (CST, 12282, 1:250 dilution) and anti-GPX4 antibody (Abcam, ab125066, 1:500 dilution), followed by Fluorescent secondary antibody (Abcam, ab150080, ab150077, 1:500). Finally, DAPI was used for nuclear staining and cell images were taken using a laser confocal microscope (Olympus, Tokyo, Japan).

### Western Blotting Analysis

Western blotting analysis was performed as previously described (Chen et al., 2014). The primary antibodies are described in “Materials” Section.

### Statistical Analysis

Data were shown as the mean ± S.E.M. for 3–5 times independent experiments. Western blotting and Fluorescence intensity was quantified through Image J software. Differences between groups were tested using one-way analysis of variance (ANOVA), followed by a Tukey-Kramer test as *post-hoc* comparison using the software Prism 8 (Chicago, USA). Differences were considered statistically significant if *P < 0.05.

## Results

### L-F001 Reduces RSL3-Induced Cytotoxicity in HT22 Cells

In our previous research, L-F001 could prevent 6-OHDA-induced cytotoxicity, and 6-OHDAwas a good ferroptosis inducer that could degrade ferritin and damage the iron homeostasis (Sun et al., 2020; Tian et al., 2020). L-F001 (1–10 μM) was incubated with RSL3 in HT22 cells to evaluate protective effects. Our results show that L-F001 reduced RSL3-induced HT22 cells’ cytotoxicity in a dose-dependent manner, and among them, 10 μM L-F001 could almost completely reverse RSL-3-induced HT22 cell injury and restore cell morphology. lipoic acid (LA) had a similar protective effect, but fasudil (Fas) had a slight protective effect (Figures 1B,C). Therefore, the ROCK inhibition of L-F001 was hardly related to the anti-ferroptosis effect. And LA group, which has the function of resisting ferroptosis (Zhang et al., 2018; Liu et al., 2020), plays an important role in the anti-ferroptosis of L-F001. Electron microscope investigation showed the shrunken mitochondria and absence of mitochondria cristae in RSL3-treated HT22 cells, and obvious improvement of mitochondrial morphology could be observed after treatment with L-F001 (Figure 1D).

### L-F001 Reduces RSL3-Induced ROS and Lipid Peroxidation in HT22 Cells

**Figure 2:**
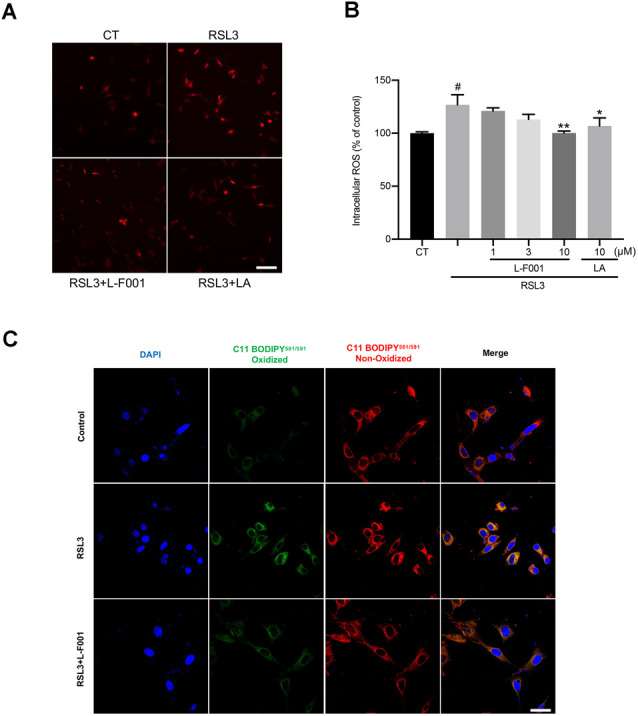
L-F001 reduces RSL3-induced ROS production and lipid peroxidation in HT22 cells. **(A)** HT22 cells were treated with 0.1 μM RSL3 alone, and then added L-F001 (1–10 μM) and LA (10 μM) at 37°C for 24 h. ROS formation was measured with a fluorescence microscope (scale bar = 50 μm). **(B)** ROS levels were measured with a fluorescence plate reader (*n* = 3). **(C)** The lipid ROS formation was monitored by a laser confocal microscope using a C11-BODIPY581/591 probe (Scale bar = 25 μm). ^#^*P* < 0.05 compared with the control group. *P < 0.05, **P < 0.01 compared with the RSL3-induced group.

We measured the intracellular ROS and lipid oxidation levels by dihydroethidium and C11-BODIPY 581/591 probes. ROS and lipid oxidation levels in HT22 cells dramatically increased under the RSL3 treatment. Pretreatment with 10 μM L-F001 significantly decreased ROS (Figures 2A,B) and lipid peroxidation levels in RSL3-induced HT22 cells (Figure 2C). Those results showed L-F001 could effectively improve the lipid metabolism process in ferroptosis.

### L-F001 Affects the Levels of Lipid Peroxidation-Related Proteins in HT22 Cells

**Figure 3:**
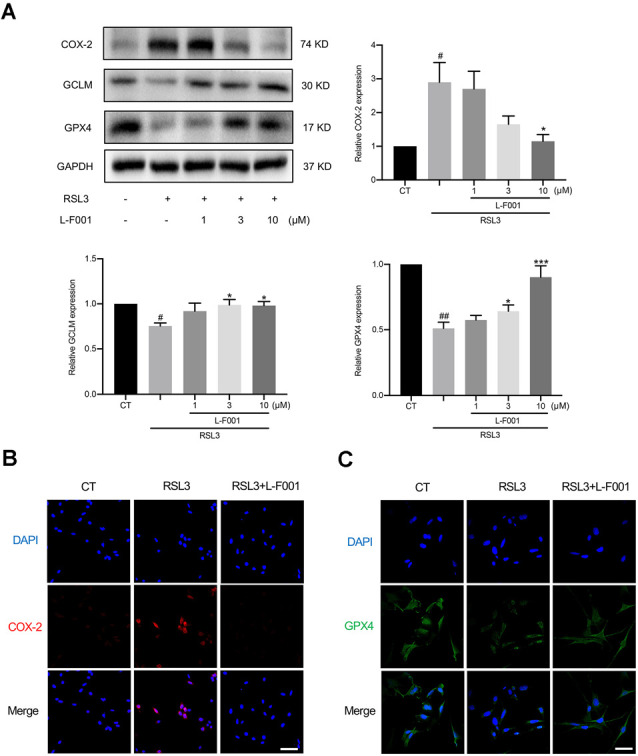
L-F001 affects the levels of lipid peroxidation-related protein proteins in HT22 cells. HT22 cells were treated with 0.1 μM RSL3 alone, and then added L-F001 (1–10 μM) at 37°C for 24 h. (**A)** The levels of COX-2, GCLM, and GPX4 were measured by Western blot, and the amount of COX-2, GCLM, and GPX4 were estimated by densitometric analysis of each protein band (*n* = 3). **(B,C)** Cells were immunofluorescence stained with anti-COX-2 and GPX4 antibodies. Nuclear counterstaining was done with DAPI (Scale bar = 100 μm). ^#^*P* < 0.05 and ^##^*P* < 0.01 compared with the control group. *P < 0.05 and ***P < 0.001 compared with the RSL3 group.

RSL3 pretreatment resulted in obvious shifts of lipid peroxidation-related proteins in HT22 cells, including cyclooxygenase-2 (COX-2; Yang et al., 2014), GCLM, and GPX4 protein levels (Seibt et al., 2019) 3 and 10 μM L-F001 pretreatment resulted in obviously reduced levels of COX-2 and restored levels of GPX4 and GCLM inRSL3-induced HT22 cells (Figure 3A). Immunofluorescence results confirmed those changes (Figures 3B,C). These data suggested that L-F001 could restore GCLM and GPX4 levels to inhibit ferroptosis in HT22 cells.

### L-F001 Reduces RSL3-Induced Impairment of Iron Homeostasis in HT22 Cells

**Figure 4:**
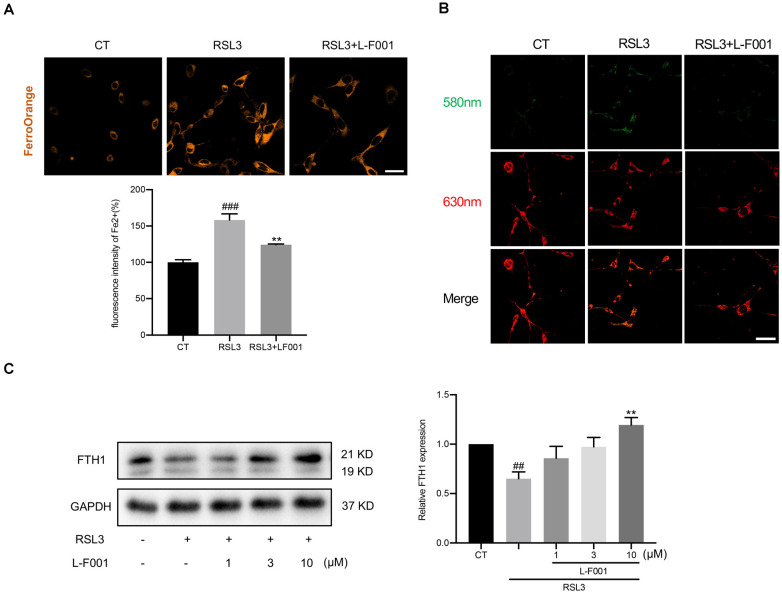
L-F001 reduces RSL3-induced impairment of iron homeostasis in HT22 cells. HT22 cells were treated with 0.1 μM RSL3 alone, and then added L-F001(10 μM) at 37°C for 24 h. **(A)** Intracellular Fe^2+^ level in HT22 cells were detected by FerroOrange probe (scale bar = 25 μm). The fluorescence intensity of the CT group was defined as 100% (*n* = 5). **(B)** Endogenous hydroxyl radicals’ level in HT22 cells were detected by Rho-Bob probe (Scale bar = 25 μm). **(C)** Protein level and analysis of FTH1 in cells (*n* = 3). ^##^*P* < 0.01 and ^###^*P* < 0.001 compared with the control group. **P < 0.01 compared with the RSL3-induced group.

Excess intracellular Fe^2+^ is a marker of ferroptosis, which triggers Fenton’s reaction to lead to membrane lipid peroxidation. We determined the Fe^2+^ by FerroOrange fluorescent probe and found that 10 μM L-F001 significantly reduced Fe^2+^ levels under RSL3 treatment in HT22 cells (Figure 4A). Meanwhile, hydroxyl radicals which were produced in Fenton’s reaction dramatically increased under the RSL3 treatment in HT22 cells. Pretreatment with 10 μM L-F001 significantly decreased hydroxyl radicals’ production in RSL3-induced HT22 cells (Figure 4B). To measure the iron storage function, we detected the level of FTH1. And Pretreatment with 10 μM L-F001 could significantly restore the FTH1 level under RSL3 treatment in HT22 cells (Figure 4C).

### L-F001 Inhibits RSL3-Induced JNK Activation in HT22 Cells

**Figure 5:**
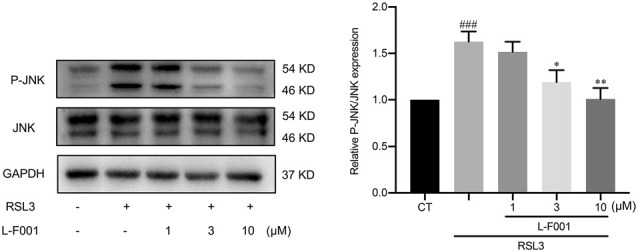
L-F001 inhibits RSL3-induced JNK activation in HT22 cells. HT22 cells were treated with 0.1 μM RSL3 alone, and then added L-F001(1–10 μM) at 37°C for 24 h. The levels of P-JNK and JNK were measured by Western blot, and the amount of P-JNK and JNK were estimated by densitometric analysis of each protein band (*n* = 3). ^###^*P* < 0.001 compared with the control group. *P < 0.05 and **P < 0.01 compared with the RSL3 group.

Lipid peroxidation also induces phosphorylation of JNK, which is involved in cellular responses to environmental stresses. To explore the potential mechanism of L-F001 inhibiting RSL3-induced ferroptosis, we detected p-JNK/JNK protein levels. p-JNK/JNK increased upon the treatment with RSL3 in HT22 cells, and 10 μM L-F001 pretreatment hindered the increase of JNK phosphorylation level (Figure 5).

## Discussion

Ferroptosis, a new type of programmed cell death is associated with iron ions and plays an important role in cerebral ischemia (Datta et al., 2020; Liu et al., 2022). L-F001 is a good neuroprotective agent with multifunctional effects such as anti-inflammation and anti-oxidative stress (Shen et al., 2015; Chen et al., 2017; Luo et al., 2017). We found that L-F001 could reduce the lipid peroxidation, impairment of iron homeostasis, and JNK activation in the RSL3-induced HT22 hippocampal neuronal cell line, providing a potential strategy apart from ROCK inhibition for treating ferroptosis-related cerebral ischemia.

MTT assay was used to evaluate cell viability in our previous studies on ferroptosis (Peng et al., 2021) and the neuroprotective effect of L-F001. L-F001 significantly rescued cell viability and showed a similar effect with LA in the RSL3-induced HT22 cells. Still, fasudil had little effect to protect RSL3-induced cell death, indicating that the protection of L-F001 was almost related to the LA group (Figures 1B,C). LA belongs to a group of B vitamins, which plays the role of coenzyme in the oxidative respiratory chain and has antioxidant activities. In recent years, it has been found that lipoic acid has an anti-ferroptosis effect, which can not only alleviate the ferroptosis of PC12 cells induced by MPP^+^ by activating PI3K/Akt/Nrf2 (Liu et al., 2021), but also alleviate the ferroptosis induced by cobalt nanoparticles (Liu et al., 2020). At the animal level, lipoic acid can significantly reduce iron overload and lipid peroxidation in P301S Tau transgenic mice (Zhang et al., 2018). Compared to LA, L-F001 has a good function of ROCK inhibition and anti-inflammation effect which were often therapeutic targets for cerebral ischemia. At the same time, L-F001 significantly recovered mitochondrial damage (Figure 1D). These findings indicated L-F001 could inhibit ferroptosis and the effect of L-F001 on ferroptosis is largely attributed to the lipoic acid group.

Lipid peroxidation is a bridge between ferroptosis and cerebral ischemia. Ferroptosis is characterized by lipid peroxidation, and the levels of lipid hydroperoxides were significantly higher in ischemic stroke patients (Zeiger et al., 2009; Yang and Stockwell, 2016). L-F001 not only reduced ROS production and lipid peroxidation (Figures 2A,B) but also affects the levels of lipid peroxidation-related protein. Meanwhile, we have found that the ROS production and cell viability after L-F001 treatment is similar with LA in RSL3-induced HT22 cells, and there was no statistical difference between the two groups in 10 μM. Therefore, we thought the LA group is the functional part of L-F001 to resist ferroptosis. And LA has been proved that it had a good 2,2-Diphenyl-1-picrylhydrazyl (DPPH) scavenging ability which represented direct scavenging ability on free radicals (Zhao and Liu, 2011). For this reason, L-F001, as a derivative of LA, might be inferred that it would also have good DPPH scavenging ability same as LA. COX-2 is a key enzyme of lipid peroxidation involved in synthesizing prostaglandins and studies have shown that COX-2 is markedly upregulated during ferroptosis, which is a downstream and suitable marker for the lipid peroxidation that occurs during GPX4-regulated ferroptosis (Yang et al., 2014). Knocking out COX-2 can reduce neuron ferroptosis and lipid peroxidation (Xiao et al., 2019). Interestingly, we found that L-F001 could decrease the level of COX-2, proving that lipid peroxidation has gone (Figures 3A,B). GCLM is a part of the first rate-limiting enzyme of glutathione synthesis, which can produce GSH, a substrate of GPX4, to resist ferroptosis (Kang et al., 2021). GPX4 is a selenium-containing membrane lipid repair enzyme, which can suppress the activation of lipoxygenase and cyclooxygenase at the nucleus and endoplasmic reticulum by reducing fatty acid hydroperoxide as activators of lipoxygenase and cyclooxygenase (Imai et al., 2017). The GPX4 inhibitor RSL3 promotes lipid peroxidation and ferroptosis (Franklin et al., 2009), which leads to GPX4 and GCLM degradation. L-F001 pretreatment could rescue RSL3-induced decrease of GPX4 and GCLM levels (Figures 3A,C). These results confirmed that L-F001 could reduce lipid peroxidation to play an anti-ferroptosis role.

Iron homeostasis is a complex process and relies on the coordination of multiple iron metabolism proteins, including the heavy and light subunit of ferritin (FTH1 and FTL), which is a protein complex that safely concentrates intracellular iron in a mineralized, redox-inactive form; transferrin, an iron-binding serum protein; ferroportin, the only known cellular iron efflux pump (Bogdan et al., 2016). Excessive Fe^2+^ is a hallmark of ferroptosis, which reacts with H_2_O_2_ to produce many hydroxyl radicals (OH•), and OH• promotes the oxidation of polyunsaturated fatty acids to hydroperoxide derivatives of lipids (LOOH) on the cell membrane (Li et al., 2008). FTH1 plays a vital role in the efflux and storage of Fe^3+^/Fe^2+^ in cells. Several previous studies elucidated that treating iron chelator deferoxamine (Van der Loo et al., 2020; Abdul et al., 2021) and promoting iron export could prevent ferroptosis damage after ischemic stroke. In the RSL3-treated HT22 cells, we observed the excessive Fe^2+^ and the downregulation of FTH1, and L-F001 can reduce excessive Fe^2+^ and restored FTH1 levels (Figures 4A–C). These results indicated that L-F001 could maintain intracellular iron homeostasis and decrease Fenton’s reaction to playing an anti-ferroptosis role.

The activation of the MAPK pathway is one of the important signs of ferroptosis (Stockwell et al., 2017), in which JNK is an important member. JNK responds to environmental stress, and cerebral ischemia can also induce the robust activation of JNK cascades, ultimately resulting in neuron death (Nozaki et al., 2001). In ferroptosis, excess lipid peroxidation products produced during the oxidation of the plasma membrane can remain active in the JNK and enhanced phosphorylation (Nagase et al., 2020; Qiu et al., 2020). And using JNK specific pharmacological inhibitors can alleviate not only ferroptosis (Fuhrmann et al., 2020) and cerebral ischemia (Sun et al., 2015; Zheng et al., 2020). L-F001 significantly reduced JNK phosphorylation (Figure 5), whose effect is reduced intracellular ROS, thus reducing the cell damage caused by JNK hyperphosphorylation. As for the other two MAPKs signaling (ERK and P38), they can all be activated by the same stimulation factors such as cytokines, neurotransmitters, hormones, cellular stress, and so on, and undergo the same changes. Many articles related to ferroptosis only detect one of them to characterize the change of ERK and P38 (Yang et al., 2021; Wang et al., 2022). In this study, we focused on the protective effects of L-F001 on ferroptosis and tentatively evaluated the JNK pathway. On the other side, our previous results indicated that L-F001 could activate Nrf-2/HO-1 signaling (Luo et al., 2017), we also found that Nrf2/HO-1 signaling is activation under RSL-3 treatment rather than downregulation (Peng et al., 2021). Furthermore, some research demonstrated that many active compounds exhibit protection through further upregulation of Nrf2/HO-1 signaling (Gou et al., 2020; Wei et al., 2021; Fu et al., 2022), compared to the already increased Nrf2 signaling. Therefore Nrf2/HO-1 signaling activation might be an important part in L-F001 anti-ferroptosis role. And in a future study, we will deeply explore the signaling pathway mechanism of L-F001 against ferroptosis such as Nrf2/HO-1, ERK, and P38.

In conclusion, due to the lipoic acid group, L-F001 is a good antioxidant and ferroptosis inhibitor, which can significantly restore RSL3-induced broken iron homeostasis, reduce lipid peroxidation, and JNK overactivation in HT22 cells. Consequently, because of its anti-ferroptosis and ROCK inhibition effect, L-F001 can potentially treat ferroptosis-related cerebral ischemia without the hypotensive response of fasudil. However, there are multiple programmed cell deaths involved in cerebral ischemia apart from ferroptosis, and the animals and clinical application need to be further verified.

## Data Availability Statement

The original contributions presented in the study are included in the article/Supplementary Material, further inquiries can be directed to the corresponding author.

## Author Contributions

WP: investigation, writing—original draft, and formal analysis. SW and JH: investigation and formal analysis. ZZ: investigation and writing—review. YY: investigation. RZ: formal analysis and validation. RP: writing—review and editing. YO: conceptualization, project administration, and supervision. All authors contributed to the article and approved the submitted version.

## Funding

This work was supported by YO from Guangzhou People’s Livelihood Science and Technology Project (No. 201903010079), Natural Science Foundation of Guangdong Province (No. 2021A1515010323), and Rural Science and Technology Commissioner Program of Guangdong Province (No. KTP2020323).

## Conflict of Interest

The authors declare that the research was conducted in the absence of any commercial or financial relationships that could be construed as a potential conflict of interest.

## Publisher’s Note

All claims expressed in this article are solely those of the authors and do not necessarily represent those of their affiliated organizations, or those of the publisher, the editors and the reviewers. Any product that may be evaluated in this article, or claim that may be made by its manufacturer, is not guaranteed or endorsed by the publisher.
